# The impact of automatic tube current modulation related settings of a modern GE CT scanner on image quality and patient dose; details do matter

**DOI:** 10.1002/acm2.14356

**Published:** 2024-04-24

**Authors:** Ioannis A. Tsalafoutas, Shady AlKhazzam, Mohammed Hassan Kharita

**Affiliations:** ^1^ Medical Physics Section Occupational Health and Safety Department Hamad Medical Corporation Doha Qatar

**Keywords:** automatic tube current modulation, CT, detectability index, image noise, mercury phantom, quality control

## Abstract

**Purpose:**

To investigate the operation principles of the automatic tube current modulation (ATCM) of a modern GE healthcare CT scanner, and the impact of related settings on image quality and patient dose.

**Material & Methods:**

A dedicated phantom (Mercury 4.0) was scanned using two of the most frequently used clinical scanning protocols (chest and abdomen‐pelvis). The preset protocol settings were used as starting points (reference conditions). Scan direction, scan mode (helical vs. axial), total beam width, tube potential (kVp), and ATCM settings were then modified individually to understand their impact on radiation dose and image quality. Regarding the ATCM settings, the SmartmA minimum and maximum mA limits, and the noise index (NI) values were varied. As surrogates of patient dose, the CTDI_vol_ and DLP values of each scan were used. As surrogates of image quality were used the image noise and the detectability index (*d’*) of five different materials (air, solid water, polystyrene, iodine, and bone) embedded in the Mercury phantom calculated with the ImQuest software.

**Results:**

The scanning direction did not have any effect on ATCM curves, unlike what has been observed in CT scanners from other manufacturers. Total beam width does matter, however, the SmartmA limit settings and kVp selection had the greatest impact on image quality and dose. It was seen that improper minimum mA limit settings practically invalidated the ATCM operation. In contrast, when full modulation was allowed without restrictions, noise standard deviation, and detectability index became much more consistent across the wide range of phantom diameters. For lower kVp settings an impressive dose reduction was observed that requires further investigation.

**Conclusion:**

SmartmA is a tool that if not properly used may increase the patient doses considerably. Therefore, its settings should be carefully adjusted for each preset different clinical protocol.

## 1 INTRODUCTION

All modern CT scanners are equipped with automatic exposure control (AEC) systems, which are more often referred to as automatic tube current modulation (ATCM) systems. The main purpose of ATCM systems is to automatically modulate the tube current, so that a certain noise level or reference image quality is obtained, depending on the diagnostic task.^1^, ^2^, ^3^ However, there are large differences between CT scanner manufacturers regarding the specifics of their mA adaptation strategy. A common characteristic is that the mapping of the attenuation characteristics of each patient (or phantom) is obtained using the scan projections radiographs (SPR) which precede the scans and calculate the mA values required during patient scanning to achieve the desired goal (henceforth referred to as mA histogram).^1^, ^2^, ^3^, ^4^

Some manufacturers use a variable level of compensation (modulation strength) to provide different levels of mA increase for obese patients and mA decrease for slim patients, preventing from both extreme mA escalations and reductions, respectively.^4^ When using this approach, it is accepted that the noise will be decreased when the scanned anatomy is thin and increased when the opposite is true. This approach is supposed to match clinical requirements more closely, since smaller patients require smoother images, as it is more difficult to differentiate organs correctly when less fat is present.^1^ Other manufacturers use a strategy that aims to achieve a constant level of noise, irrespective of the differences in size and density between different patients and between different anatomic regions within the same patient. However, as a safeguard against extreme mA variations with very thin or very obese patients, they limit the minimum and maximum mA values that can be used by the ATCM system.^1^

A typical example of the second strategy is the  General Electric Healthcare (GE) CT scanners, where the target image quality is determined by the noise index (NI): the smaller the NI value is, the better the image quality and the larger the dose. It must be noted that according to the GE's CT scanner documentation “The noise index value will approximately equal the standard deviation in the central region of the image when a uniform phantom (with the patient's attenuation characteristics) is scanned and reconstructed using the standard reconstruction algorithm.” In GE scanners, when the ATCM modulation is activated by selecting the ‟SmartmA‘‘ option (enables longitudinal collimation and angular collimation), apart from the preset NI value, the preset minimum and the maximum mA values allowed are also shown. The CT scanner user can either use the preset values as is or modify them to promote or suppress the mA modulation. For example, if the user increases the mA value for the minimum mA limit, the use of smaller mA values will be prevented, irrespectively if these are in accordance with the mA histogram obtained from the SPR, suppressing in this way the mA modulation.

Regarding the ATCM systems, the AAPM TG233 report^4^ analyzes in depth the ATCM system specifics, and reviews old and new image quality indices that can be used for image quality assessment in conjunction with the evaluation of ATCM systems. This report sets no performance guidelines nor pass‐fail criteria, but instead proposes assessment methods for approaching the operational performance testing of new CT systems. These methods can improve the understanding and utilization of the technologies available in each CT scanner and potentially will be used to compare CT scanners and examination protocol settings in terms of their performance in clinical conditions.^4^ The APPM TG233 report does not endorse any particular commercial phantom, but rather explores the utility of some commercially available phantoms, including the Gammex Mercury 4.0 phantom. The methods described in the AAPM TG233 report^4^ have been extensively used in numerous papers which use various versions of the Mercury phantom, the ACR 464 CT phantom but also other phantoms.^5^, ^6^, ^7^, ^8^, ^9^, ^10^, ^11^

**FIGURE 1:**
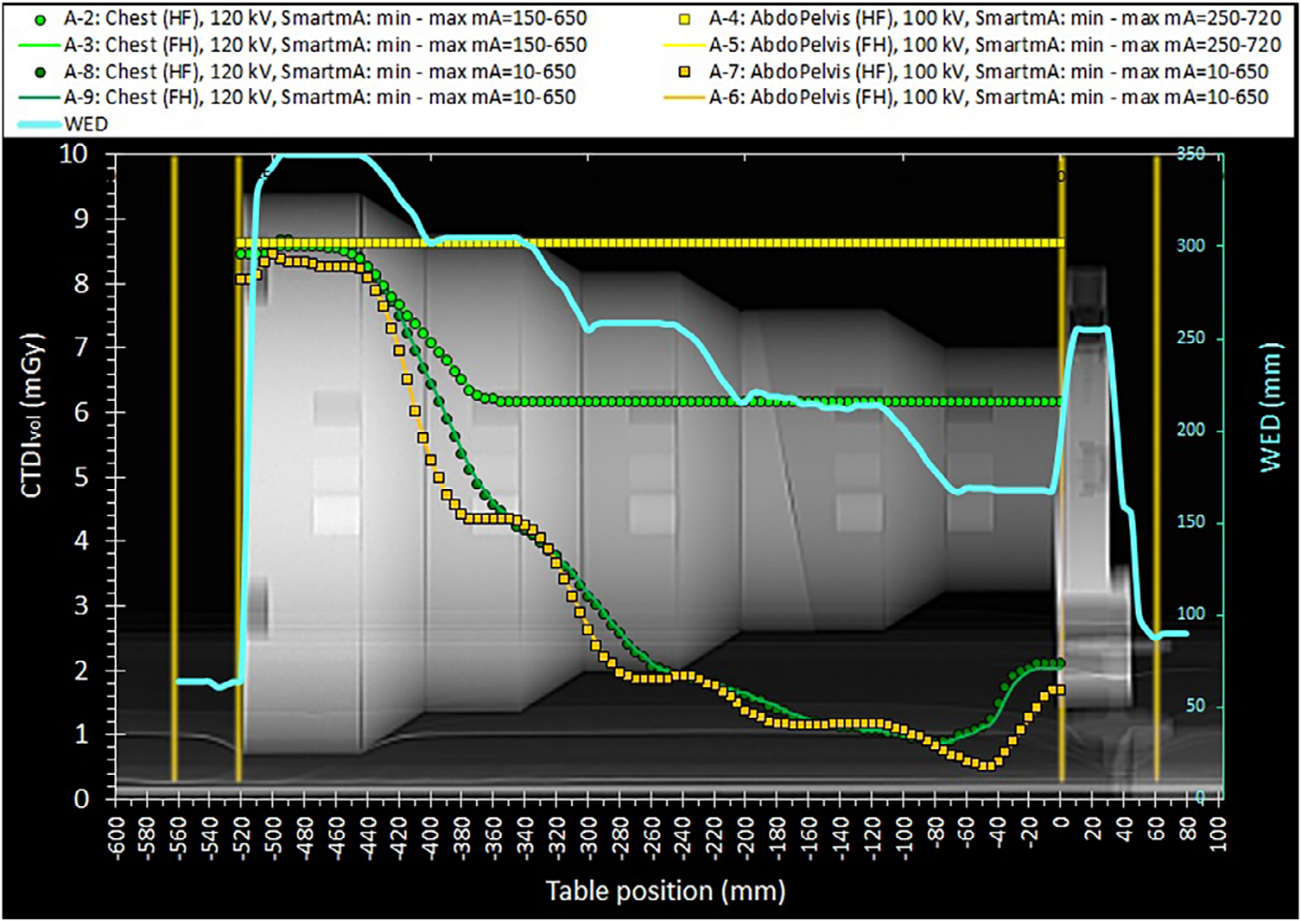
The CTDI_vol_ modulation curves obtained with the Chest and AbdoPelvis acquisition protocols (for both HF and FH scanning directions) using the preset SmartmA setting and the modified minimum SmartmA value to allow full mA adaptation. The pair of orange lines closer to the phantom ends is the scan length of the majority of spiral scans (52 cm).

In a new Hammad Medical Corporation hospital a modern CT scanner was recently installed (Revolution CT, GE Healthcare, Chicago, Illinois, USA). During the commissioning procedure and at a later stage, along with the standard quality control (QC) tests performed, a number of scans were performed using the Mercury phantom to investigate certain scanner features related to the ATCM system operation, using as a starting point the preset settings of the Chest and Abdomen‐Pelvis examination protocols.

## 2 MATERIALS AND METHODS

The main material of the Mercury 4.0 phantom is virgin ultra‐high molecular weight polyethylene (density = 0.93 g/cm^3^ and CT# = −90 HU) and is made up of five cylinders and four cone‐rings (tapered transitional sections between the cylinders) whose dimensions are described in Table 1. The five cylinders of the phantom contain five rods (targets) of the same diameter (2.5 cm) and length (3 cm), positioned at a distance 4.5 cm around the phantom's central axis. These are constructed by HE CT Solid Water® (CT# = 0 HU), bone (CT# = 910 HU), polystyrene (CT# = −40 HU), Iodine (10 mg/mL) (CT# = 245 HU), and air (CT# = −985 HU).

**TABLE 1:** Description of the Mercury 4.0 phantom.

Phantom regions	Shape and number	Diameter (cm)	Length^a^ (cm)	WED (mm) [nominal]
Section 0	Handle	–	–	max 256
Section 1	Cylinder #1	16	7	168
Section 2	Cone ring #1	16–21	4	169–215
Section 3	Cylinder #2	21	9	215 (222^b^)
Section 4	Cone ring #2	21–26	4	217–255
Section 5	Cylinder #3	26	6	259
Section 6	Cone ring#3	26–31	4	260–298
Section 7	Cylinder #4	31	6	305
Section 8	Cone ring #4	31–36	4	302–346
Section 9^c^	Cylinder #5	36	7	351

*Note*: The water equivalent diameter was calculated by the software (ImQuest v 7.2) which is used for the automatic evaluation of image quality.

^a^ In the cylindrical sections, 3cm contain the 5 rods and the rest is uniform.

^b^ Maximum WED observed at the region of the solid water ramp used for z‐axis resolution measurements.

^c^ A handgrip is engraved within the phantom.

The phantom was positioned with its smallest diameter closest to the CT gantry. According to the protocols’ preset settings, two SPRs are required, one anteroposterior (AP) and one lateral (LAT) using 120 kV, to be able to use the SmartmA ATCM option. The scan length of the SPRs was selected to allow the coverage of the whole phantom plus about 10 cm beyond both ends of the phantom. In the following acquisitions two planned scan lengths were used: the one was that used in most of the scans, was limited from the beginning of the phantom's smallest diameter (16 cm) cylindrical section to the end of the largest diameter (36 cm) cylindrical sections (henceforth referred to as limited scan length), and the other that was extending beyond the phantom boundaries by about 4 cm (henceforth referred to as extended scan length), which was used for only a few scans.

As basis for all acquisitions two of the most often used clinically examination protocols were employed; those used for scanning the chest (CT Chest routine plain, henceforth referred to as Chest) and the abdomen‐pelvis (CT AbdoPelvis, henceforth referred to as AbdoPelvis) anatomic areas. The preset settings of the Chest and the AbdoPelvis examination protocols are shown in Table 2. The scans acquired can be clustered in three groups: Group A, B, and C. In group A there are three subgroups. In the first subgroup (see Table 3: A‐2 to A‐5), the SmartmA preset settings for minimum and maximum mA limits shown in Table 2 were used. Two different scanning directions, that is, from head‐to‐feet (HF) and from feet‐to‐head (FH), were used to investigate if the scan direction affects the ATCM patterns.

**TABLE 2:** Preset settings for the Chest and AbdoPelvis acquisition protocols (scan field of view = 50 cm).

Protocol	Scan mode	Total beam width (mm)	Collimation (mm)	pitch	kVp	Rotation time (s)	Convolution kernel	Reconstruction slice thickness (mm)	ATCM: SmartmA min mA–max mA	NI	ASIR‐V^a^ level (%)
Chest	Helical	80	128 × 0.625	0.992	120	0.6	CHST	5	150–650	12	50
AbdoPelvis	Helical	40	64 × 0.625	0.984	100	0.8	Standard	5	250–720	12	60

^a^ ASIR‐V is a trademark for the GE's proprietary iterative reconstruction algorithm.

**TABLE 3:** Description of the scan acquisitions and the reconstructed series presented in this study.

ID‐Code	Protocol	Scan direction	Scan mode	Pitch	Total beam width (mm)	SmartmA (min mA–max mA)	NI	Rotation time (ms)	kVp	CTDI_vol_ (mGy)	DLP (mGycm)	Scan length (cm)
A‐2	Chest	HF	Helical	0.992	80	150–650	12	600	120	6.66	387.06	52.0
A‐3		FH								6.65	386.98	
A‐4	AbdoPelvis	HF		0.984	40	250–720		800	100	8.58	471.24	
A‐5		FH								8.58	471.24	
A‐8	Chest	HF	Helical	0.992	80	10–650	12	600	120	3.83	222.45	52.0
A‐9		FH								3.81	221.75	
A‐7	AbdoPelvis	HF		0.984	40			800	100	3.44	188.78	
A‐6		FH								3.43	188.26	
A‐11	Chest	HF	Helical	0.984	40	10–650	12	600	120	3.07	199.24	58.0
A‐10				0.992	80					3.56	242.73	
A‐13			Axial	–	40					3.01	192.90	63.5
A‐14				–	80					4.50	296.30	65.5
A‐15				–	5					4.25	257.21	60.0
B‐6	Chest	HF	Helical	0.984	40	10–650	12	1000	140	3.56	195.63	52.0
B‐2									120	3.19	174.99	
B‐5									100	2.62	143.89	
B‐4									80	1.78	97.75	
B‐3									70	1.19	65.18	
B‐7	Chest	HF	Helical	0.984	40	100–600	12	500	Auto kV: GE Thorax (Adult) = 80^a^	1.73	95.10	52.0
B‐8						10–600				1.61	88.74	
B‐14	AbdoPelvis	HF	Helical	0.984	40	10–500	12	1000	140	2.55	140.05	52.0
B‐15									120	2.29	125.64	
B‐13									100	1.81	99.57	
B‐12									80	1.20	65.88	
B‐11									70	0.79	43.39	
B‐9	AbdoPelvis	HF	Helical	0.984	40	250–600	12	500	Auto kV: GE AbdPelvis (Adult) = 80^b^	2.74	150.33	52.0
B‐10						10–600				1.36	74.83	
C‐6	Chest	HF	Helical	0.984	40	10‐650	16	1000	120	1.65	90.80	52.0
C‐5							14			2.29	125.72	
C‐4							10			5.45	299.52	
C‐3							8			9.85	541.08	
C‐2						10‐630	6.9^6^			17.91	983.69	

^a^ The noise index automatically changed to 12.4.

^b^ The noise index automatically changed to 11.5.

In the second subgroup (see Table 3: A‐6 to A‐9), the above four scans were repeated after modifying the SmartmA settings to 10 mA for the minimum mA and to 650 mA for the maximum mA settings (henceforth referred to as modified SmartmA settings), to allow full modulation with no restrictions, in order to see how this will affect the dose and the image quality. In the third subgroup (see Table 3: A‐10 to A‐15), the Chest protocol, the HF direction, and the modified mA Smart scan settings were used, and the only parameters changing were the scan mode (helical or axial) and the total beam width (40‐ and 80‐mm for axial and helical, and additionally 5 mm for the axial mode), to investigate how the total beam width can affect the dose and the image quality. In this subgroup scans (see Table 3: A‐10 to A‐15), the extended scan length was used to include the handle section and air sections in both phantom ends, to investigate how the ATCM system responds when moving from the air to the phantom and vice versa.

In the second group the Chest (see Table 3: B‐2 to B‐8) and AbdoPelvis (see Table 3: B‐9 to B‐15) protocol were used, with modified SmartmA settings and 40 mm total beam width and pitch 0.984 for both protocols, and the only parameter changing was the manually selected kV (70, 80, 100, 120, and 140 kV) to investigate how the kVp selection can affect the dose and the image quality. For each protocol two additional scans were made using the automated tube voltage selection (ATVS) option and the preset and modified SmartmA settings. In this GE model the ATVS mode is referred to as Auto prescription kV mode and it is combined with the auto prescription profile (for specific examinations) and henceforth will be referred to as Auto kV (note that the kVp selection, manual or auto, remains constant during the scan). In the third group (see Table 3: C‐2 to C‐6), the Chest protocol, the chest protocol with modified SmartmA settings, beam width 40 mm, and pitch 0.984 was used, with different NI settings, to investigate how the NI can affect the dose and the image quality.

The ImQuest v7.2 software (Clinical Imaging Physics Group, Department of Radiology, Duke Health, ImQuest@duke.edu) that has been produced for evaluation of image quality with the Mercury phantom (and the ACR 464 phantom) according to the methodology described in the AAPM TG233 report,^4^ was used in this study for producing: a) the water equivalent diameter (WED) of the Mercury sections with respect to the Z‐axis value (table position), b) various image quality indices, including the detectability index (*d*’) which is the only image quality metric produced by ImQuest that will be presented in this paper.

While a detailed description of *d’* can be found elsewhere,^4^ it can be said in brief that *d*’ is calculated in the Fourier domain using the Non‐Prewhitening Model Observer with Eye Filter (NPWE), the noise power spectrum (NPS), and the task transfer function (TTF), to simulate human observer performance in clinical interpretation tasks, as for example the detection of circular objects of different size and contrast in the presence of different noise levels. Thus, the *d*’ values obtained from the ImQuest software can be used as a surrogate of the overall image quality, and the larger the *d* ’ the better image quality is. ImQuest is normally able to automatically calculate all image quality parameters, but for the specific data set, it failed completely to evaluate all the image series obtained using 70 and 80 kV. For the series acquired with 100 kV, it failed to calculate the *d*’ values for the phantom section with the largest diameter. For this reason, a semi‐automatic image quality evaluation method had to be used for all image series. For each scan series, the ROIs for calculating the noise power spectrum (NPS) and task transfer function (TTF) were manually selected, as well as the CT images in each phantom section from where the NPS and the TTF values were first calculated, in order to calculate the *d*’ values. In this way, the *d*’ values for all phantom sections were calculated section by section, and then the *d*’ values for all sections (along other image quality related metrics) were exported to a comma‐separated values (csv) file format.

A free software (ImageJ 1.53a, https://imagej.nih.gov/ij/index.html) and in‐house build macros were also used to read the CT number and standard deviation (SD) of all reconstructed CT images. A free software (DICOM Info Extractor^5^) was used to read the DICOM headers of the reconstructed CT images, to derive information about the scanning and reconstruction parameters of each image, including the table position values and the respective mA and rotation time values, by which we calculated the respective mAs and estimated the CTDI_vol_ values. This is because, GE in contrast to other CT scanner manufacturers, does not store in the DICOM data of each image the CTDI_vol_ value nor the correct mAs value. The mAs value given in the DICOM tag 0018,1152 is the truncated value of the product of mA and rotation time value, divided by the total beam width and multiplied by the reconstructed slice thickness. To give an example, the series with ID = A‐4 (beam width = 40 mm, reconstructed slice thickness = 5 mm), where practically constant mAs were used, for all images the mAs tag value was 25 whereas the product of mA and exposure/revolution time was 199 (199 mAs × 5 mm/40 mm = 25 mAs.)

The method described elsewhere^12^ was used to calculate the CTDI_vol_ (= CTDI_w_) values first from the axial scans with total beam width 40 and 80 mm, so as the calculated average CTDI_vol_ and DLP agree with the displayed values (within ± 2%). Then this was adapted for the helical scans, considering that part of the DLP is due to overscanning (also referred to as overranging), which results in up to one additional rotation at each end of the planned scan length, for which no images are reconstructed. As surrogates of patient dose in each scan, the respective CTDI_vol_ and DLP values were used. To compare the results of the ATCM for each different phantom section, the CTDI_vol_ values were used when kVp, pitch or rotation time were different, but mA values were preferred in cases where kVp, rotation time and pitch were identical (e.g., A‐2 to A‐15 and B‐2 to B‐8).

The information regarding the technical parameters used for all scans performed and the resulting CTDI_vol_ and DLP values are given in Table 3. In the following, the ATCM modulation curves will be depicted in terms of CTDI_vol_ or mA versus table position of each reconstructed CT image (using the DICOM data). Unlike the mA modulation curves, the CTDI_vol_ curves represent the dose modulation, and account for the effects of kVp, rotation time, pitch, and dose efficiency. In the respective graphs, along these CTDI_vol_ or mA modulation curves (henceforth referred to as modulation curves) an additional curve was depicted, namely the WED (secondary Y‐axis) versus table position. All these curves were superimposed on scan projection radiograph (SPR) images, where the positions of the first and the last image positions of each scan series are also depicted (with orange‐colored vertical lines), to be able to observe the dose modulation (primary Y‐axis), with respect to the different phantom sections. In this way, the results of ATCM modulation were visually studied in terms of simultaneous variations of CTDI_vol_ or mA and WED with respect to position of the CT image within the phantom.

## 3 RESULTS

**FIGURE 2:**
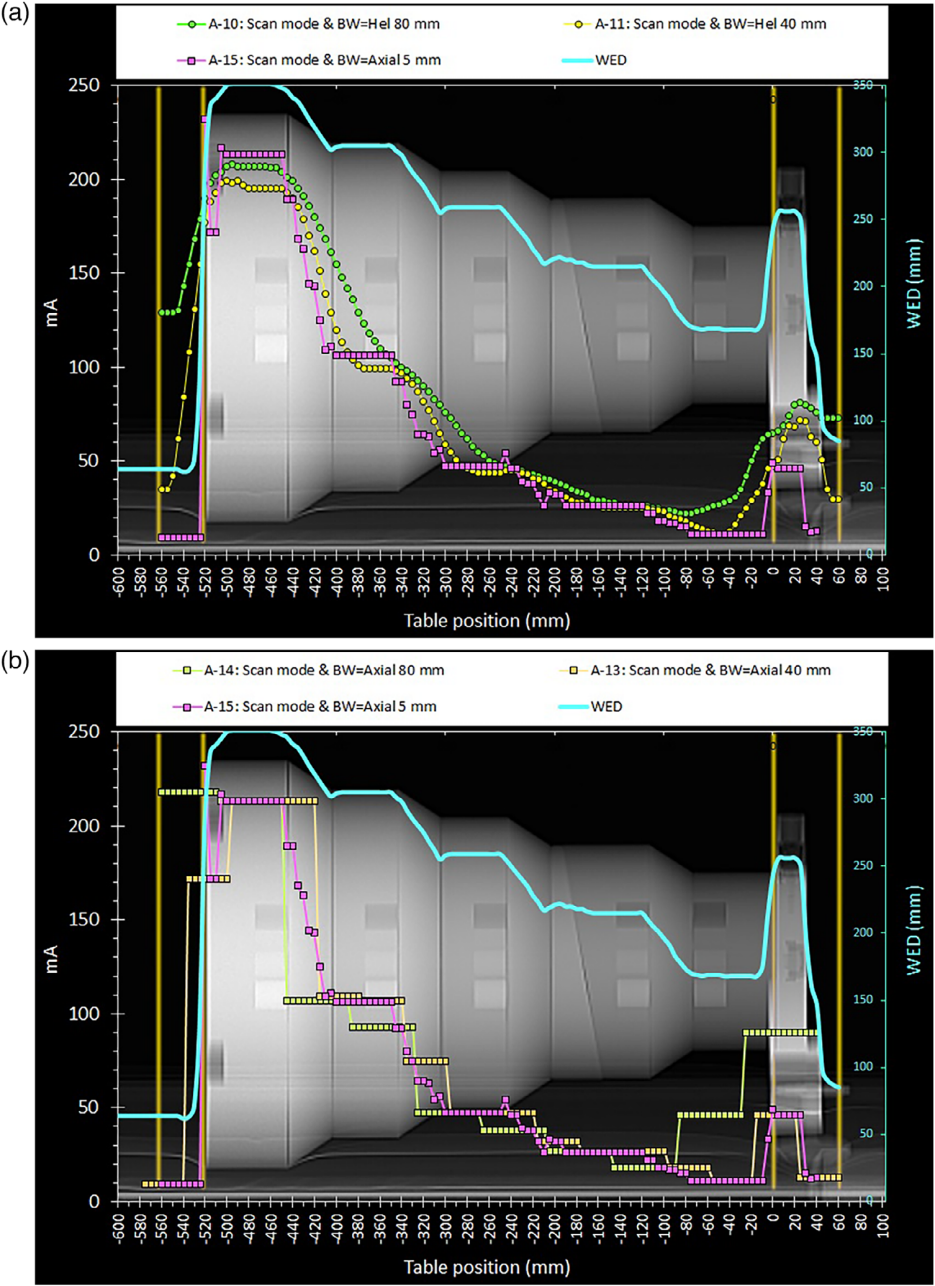
(a) The mA modulation curves obtained with the Chest acquisition protocol, using the modified SmartmA settings and different total beam widths and scanning modes. The outer orange lines denote the planned scan length of the A‐11 and A‐12 spiral scans (58 cm), where the air gaps were included to study how the modulation works at the steep phantom‐air interfaces. (b) The mA modulation curves obtained with the Chest acquisition protocol, using the modified SmartmA settings and different total beam widths in axial mode. As seen from the first and last points of each mA modulation curves the different total beam widths resulted in different planned scan lengths.

**FIGURE 3:**
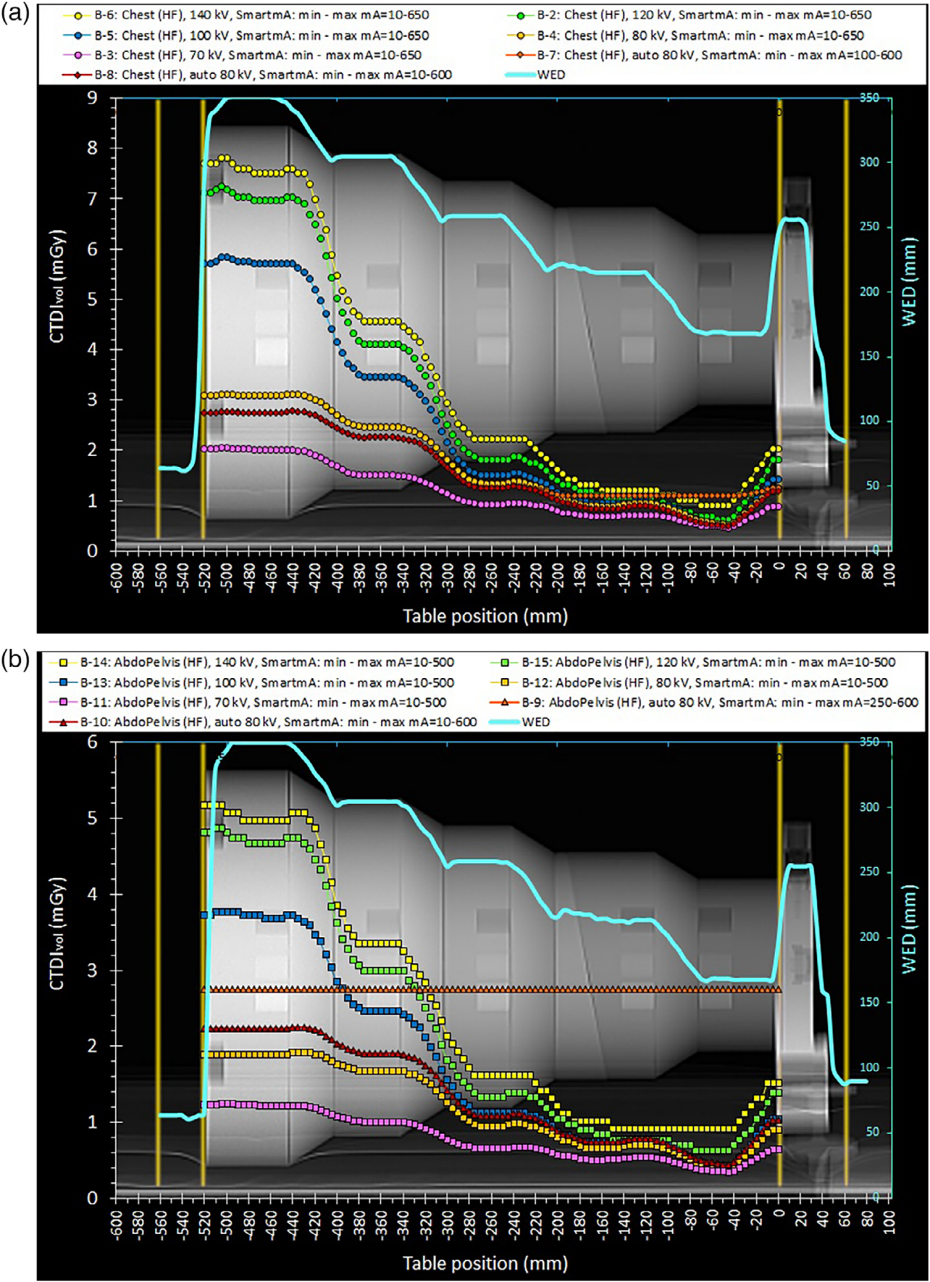
(a) The mA modulation curves obtained with the Chest acquisition protocol using different kVp settings and a rotation time of 1 s. For the Auto kV mode selection, two scans were made: one using the preset and the other using the modified SmartmA settings (minimum mA set at 10 mA). (b) The mA modulation curves obtained with the AbdoPelvis acquisition protocol using different kVp settings and a rotation time of 1 s. For the Auto kV mode selection, two scans were made: one using the preset and the other using the modified SmartmA settings (minimum mA set at 10 mA).

**FIGURE 4:**
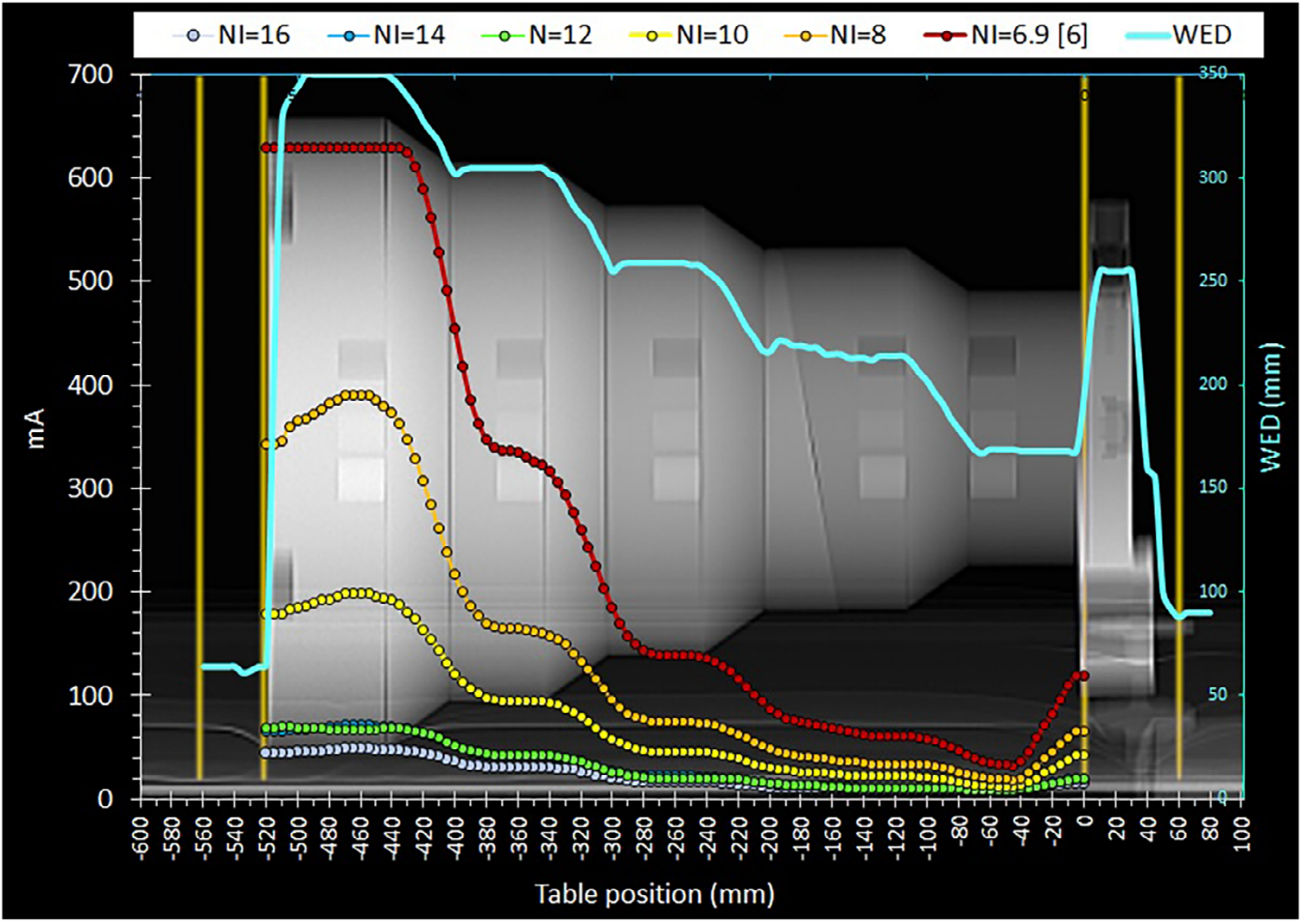
The mA modulation curves obtained with the Chest acquisition protocol using different NI values settings. The rotation time was adjusted at 1 s and the modified smartmA settings were used.

The CTDI_vol_ or mA modulation patterns resulting from the selected scan settings are shown in Figures 1, 2, 3, 4. For all these figures, the WED curve shown (secondary Y‐axis), was produced by the ImQuest software using the reconstructed CT images obtained from each scan series, for 120 kV. While there was practically no difference between the WED curves obtained from different scan series using 120 kV, small differences were observed for scans performed using different kVp.

Regarding Figure 1, there are three points that worth to be noted. First, the CTDI_vol_ curves are practically the same for both scanning directions, and this is also valid for the CTDI_vol_ and the DLP values given in Table 3 (A‐2 to A‐8), unlike what has been observed in a previous study.^11^ Second, for the preset SmartmA settings for the AbdoPelvis protocol (A‐4 and A‐5) no modulation is observed along all phantom sections, as if manual mA were used, due to the very large preset value for minimum mA (250 mA) of SmartmA. For the Chest protocol (A‐2 and A‐3) where the preset minimum mA value is lower (150), modulation is observed only after the first half of the 31 cm diameter phantom section. When the SmartmA settings were modified to allow a free mA modulation according to the phantom thickness (WED), the AbdoPelvis protocol (A‐6 and A‐7) ended up having smaller CTDI_vol_ and DLP values than the Chest protocol (A‐8 and A‐9), as can be seen in Table 3. Third, the CTDI_vol_ modulation curves obtained for AbdoPelvis with the modified SmartmA settings (A‐6 and A‐7) seem to better follow the WED variations that the respective curves for the Chest protocol (A‐8 and A‐9), presumably due to the smaller total beam width of AbdoPelvis protocol compared with that of the Chest protocol (40 mm vs. 80 mm).

Regarding the effect of total beam width on ATCM operation, both Figures 2(a) and (b) confirm what was observed in Figure 1, regarding the better adaptation to the phantom thickness variations when using smaller beam widths, either with axial or helical scan mode (the 5 mm modulation curve is included in both figures to serve as comparison standard). The better adaptation can also be assessed by way the mA values are reduced at the phantom‐air interfaces, where the 80 mm beam width is clearly inferior to the 40 and 5 mm curves (but superior regarding scanning speed).

It should be first noted that the 5 mm axial scan required a very long scanning time (about 5 min) compared to the scan time required for helical and axial scans using 40‐ or 80‐ mm total beam width (less than 30 s). Therefore, despite the apparently better adaptation observed with the 5 mm total beam width scan, using such a small beam width is impractical. Furthermore, it does not reduce DLP, since as can be seen in Table 3, the DLP of the 5 mm beam was larger than both helical scans and the axial scan with 4 cm total beam width, unlike what was probably expected when looking Figure 2(a),(b). This is because the dose efficiency of the 5 mm collimation (59%) is smaller compared to that of the 40 mm (89%) and 80 mm (94%) x‐ray beam, and this results in an increased CTDI_vol_ value. This is a typical example that demonstrates that mA modulation curves do not show the full picture regarding dose modulation, when different collimations, pitch values, kVp or rotation times are involved. It should also be noted that the 5 mm modulation beam exhibited small mA maxima at all interfaces, with a larger maximum appearing at the interface of the largest diameter cylinder and air.

In Figure 3(a),(b), are shown the CTDI_vol_ modulation curves obtained with the Chest (B‐2 to B‐8) and the AbdoPelvis (B‐9 to B‐15) protocols, using different manual and automatic kVp selections. As can be seen in both figures and Table 3, the smaller the kVp selection, the lower CTDI_vol_ and DLP values were. Even for the largest diameter section, 70 kV seemed to be the best choice in terms of patient dose, something that was a little surprising, but it was thought that it would also result in a distinct impairment of image quality (to be discussed later). The automatic kVp mode selected the 80 kV, no matter whether the SmartmA settings were the preset ones or the modified values, for both Chest and AbdoPelvis protocols. The high minimum mA values of the preset SmartmA settings in the case of the Chest protocol (100 mA) resulted in practically no modulation in the two smaller diameter phantom sections, while in the case of the AbdoPelvis (250 mA) resulted in zero modulation along the whole phantom length. Note that, to avoid any “too high mA” warnings that could appear for the lower manual kVp selections, the rotation time was adjusted to 1 s, whereas for the Auto kV acquisitions, the rotation time had the default value of 0.5 s. Also note, that for the Chest Auto kV the NI was automatically adjusted to 12.4 while for the AbdoPelvis the Auto kV curve the NI was automatically adjusted to 11.5. This gave rise to small differences between the modulation curves for the manual selected 80 kVp and the respective Auto kVp (obtained both with the modified smart mA settings).

**FIGURE 5:**
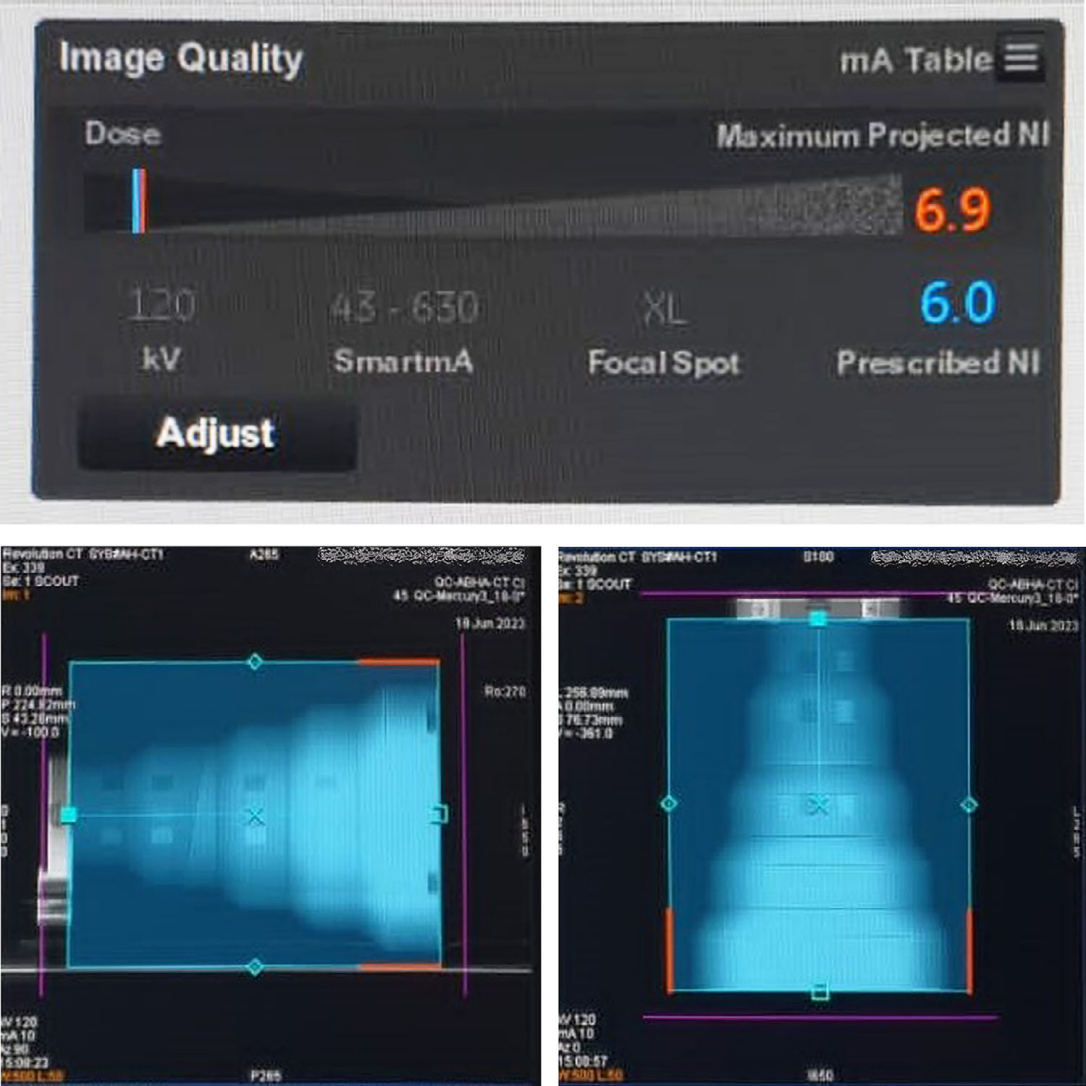
For the smallest NI selected (NI = 6), a predicted NI value of 6.9 was given, due to limitations on the maximum mA value to 630 mA that had to be imposed after a generator projected tube heat warning.

In Figure 4, the CTDI_vol_ modulation curves obtained with the Chest protocol using different NI settings are shown. It must be noted that when the NI was set to 6, there were warnings (pop‐up texts and graphs) to inform us that the target NI of 6 was not feasible and the predicted NI would now be 6.9, most probably referring to the thickest part of the phantom. Indeed, the locations where the GE system failed to achieve the initial target NI values, were shown on the screen with red side lines, as shown in Figure 5. Indeed, in Figure 4 it can be seen that for NI = 6 (6.9), the mA maxed out at 630 mA, a few cm before the phantom section with the largest diameter, as predicted by Figure 5.

Regarding the image quality evaluation, apart from the fact that the semi‐automatic mode was very time‐consuming, the *d*’ values obtained with the semi‐automatic mode shown in Table 4, were in most cases similar to those obtained automatically. For all scan series, the *d*’ values of each phantom section were larger for bone, followed by those for air, iodine, water, and polystyrene. In general, when comparing *d*’ values between different scan series for the same section it must be remembered that for the same kVp, convolution kernel and ASIR‐V level, larger CTDI_vol_ values result to either smaller noise values or/and larger TTF values and therefore larger *d*’ values, since *d*’ is a function of TTF and NPS and increases for larger TTF values and smaller values of noise.

**TABLE 4:** Results of detectability values of the five different rod materials present in the five different nominal diameters (values in parenthesis, in mm) of the Mercury phantom, obtained using the ImQuest software.

	Air					Water					Bone					Polystyrene					Iodine				
ID	*d*' (160)	*d'* (210)	*d'* (260)	*d'* (310)	*d'* (360)	*d'* (160)	*d'* (210)	*d*' (260)	*d'* (310)	*d'* (360)	*d'* (160)	*d*' (210)	*d'* (260)	*d'* (310)	*d'* (360)	*d'* (160)	*d'* (210)	*d'* (260)	*d'* (310)	*d'* (360)	*d'* (160)	*d*' (210)	*d'* (260)	*d'* (310)	*d'* (360)
A‐2	493	301	214	140	108	53.7	27.5	19.3	11.4	10.1	576	337	237	153	119	28.1	14.5	9.5	5.4	4.1	197.2	115.6	80.7	51.5	41.4
A‐3	513	318	219	145	108	54.5	30.4	17.5	11.9	9.3	590	355	240	157	118	28.9	14.2	10.3	6.0	5.4	205.9	123.2	83.5	53.6	41.0
A‐4	**718**	382	301	191	122	**93.5**	43.2	32.4	20.4	12.9	**969**	504	388	243	155	**45.0**	19.7	14.8	8.5	5.7	**358.8**	184.7	142.3	89.1	55.8
A‐5	642	398	293	194	118	83.8	45.9	31.8	20.5	12.8	865	525	372	249	148	39.1	20.3	14.2	9.5	7.2	321.8	192.8	138.2	91.7	55.3
A‐8	274	151	143	133	115	28.8	15.0	12.5	9.7	8.4	315	168	156	146	126	13.5	7.6	6.9	6.0	4.7	108.3	59.5	53.0	49.8	43.9
A‐9	263	151	145	135	108	27.9	14.1	12.4	10.6	8.5	303	169	161	148	119	14.4	7.1	7.4	5.9	5.9	106.2	58.4	54.8	49.5	42.5
A‐7	256	169	155	136	117	32.7	19.0	17.6	14.1	12.6	336	221	196	171	149	14.1	7.6	6.6	5.9	4.9	124.0	81.6	71.1	63.1	55.1
A‐6	239	175	159	144	119	30.7	19.6	16.6	15.4	12.6	319	228	198	183	152	13.0	8.0	6.6	5.6	6.7	119.0	84.2	74.5	66.1	56.8
A‐11	202	147	135	118	110	22.0	13.7	11.1	9.8	9.9	226	165	147	129	122	8.4	5.6	6.0	5.0	5.8	81.0	57.7	49.5	44.0	39.5
A‐10	265	149	147	137	108	29.3	14.0	12.2	10.5	9.4	305	166	160	149	117	15.0	6.4	6.9	6.3	5.9	107.9	57.5	55.0	51.4	41.6
A‐13	167	147	124	122	104	15.4	12.8	10.7	9.6	9.3	162	166	135	133	114	9.6	6.3	5.3	5.1	5.4	65.2	56.6	47.1	44.4	39.4
A‐14	311	147	160	118	110	32.9	13.1	13.7	9.4	9.8	363	163	175	128	120	17.7	6.9	6.8	4.9	5.9	127.3	55.7	60.2	43.6	40.9
A‐15	147	112	112	111	100	15.2	11.0	9.8	9.3	8.4	168	127	123	122	110	7.4	5.8	5.2	4.8	4.6	58.5	43.7	42.7	41.4	37.9
B‐6	223	129	134	125	106	20.3	10.8	9.5	8.9	8.5	238	132	133	124	106	12.2	6.3	5.3	5.5	5.4	77.0	42.2	43.3	40.5	34.3
B‐2	222	145	138	122	98	23.7	13.4	11.9	10.1	8.1	256	162	151	132	108	12.2	7.3	6.7	5.6	5.2	89.0	55.8	53.4	45.9	37.3
B‐5	203	134	126	112	92	26.3	14.1	12.6	11.2	9.9	265	170	158	139	114	10.8	6.6	6.0	5.4	4.3	98.6	62.4	58.8	50.2	41.7
B‐4	194	129	118	90	57	29.9	17.3	15.1	11.3	8.2	307	199	180	136	85	11.4	6.5	6.5	4.9	3.4	119.8	77.8	71.3	54.1	34.3
B‐3	170	118	85	61	43	30.8	18.7	13.9	8.7	6.5	300	202	142	100	68	9.1	6.9	4.1	3.3	2.9	120.6	81.6	58.4	41.3	28.9
B‐7	246	150	106	78	58	38.2	20.2	13.5	9.6	7.2	389	232	161	119	85	14.5	7.7	5.2	3.6	3.8	153.1	91.6	63.6	47.0	33.2
B‐8	192	126	115	90	57	28.9	16.9	14.5	11.4	7.8	304	197	174	134	85	11.2	6.7	6.0	5.0	3.0	117.4	76.6	70.6	52.3	33.8
B‐14	249	138	133	119	96	23.2	11.1	9.2	8.2	7.5	267	145	134	120	98	13.9	5.1	5.6	5.1	5.1	87.9	47.3	44.0	38.2	31.2
B‐15	242	147	136	119	91	26.6	13.2	12.2	10.1	8.0	293	167	153	131	103	14.4	7.5	5.1	5.0	5.1	102.5	57.7	52.4	45.1	35.3
B‐13	220	141	119	105	80	29.1	16.0	13.5	10.5	8.9	289	184	151	133	102	12.0	6.7	5.1	4.1	4.1	106.7	67.5	56.5	48.7	37.2
B‐12	199	130	104	79	48	32.2	18.0	14.4	10.0	6.0	320	206	162	121	73	11.8	6.5	5.6	3.4	3.1	126.4	82.6	64.0	48.4	29.2
B‐11	116	114	79	54	30	21.3	18.7	12.8	8.1	4.9	261	204	133	92	49	9.7	5.6	3.8	2.0	1.5	131.0	82.8	56.9	37.2	20.4
B‐9	432	217	164	101	61	68.5	31.2	22.0	13.3	8.5	715	353	254	158	94	27.7	11.5	8.3	5.5	3.3	280.8	138.9	101.9	62.7	38.1
B‐10	207	133	109	84	53	34.0	18.7	14.9	10.9	7.1	337	213	170	130	81	12.2	6.5	4.3	4.3	3.0	132.9	83.6	67.6	51.7	32.8
C‐6	193	123	108	89	73	20.7	10.9	9.7	7.4	6.2	220	139	116	96	81	9.5	6.2	4.1	3.5	3.9	77.9	46.6	39.5	32.0	28.0
C‐5	216	123	118	102	85	21.2	10.8	9.9	8.4	7.4	239	137	129	111	94	12.9	6.1	5.3	4.7	3.4	75.4	48.1	44.6	37.7	32.2
B‐2	222	145	138	122	98	23.7	13.4	11.9	10.1	8.1	256	162	151	132	108	12.2	7.3	6.7	5.6	5.2	89.0	55.8	53.4	45.9	37.3
C‐4	251	188	164	162	138	26.4	18.5	12.4	12.9	12.6	288	213	181	177	153	14.7	8.3	8.0	6.8	7.6	101.1	74.3	63.6	60.8	52.7
C‐3	338	226	224	206	195	36.3	20.2	19.0	17.3	18.3	389	255	246	227	216	19.1	10.4	8.9	9.8	10.8	136.7	87.8	84.3	76.9	73.6
C‐2	416	312	298	293	252	44.6	29.1	25.6	25.3	22.0	474	352	326	317	279	23.9	15.0	13.9	13.5	13.9	166.3	121.4	112.9	108.5	95.3

*Note*: The maximum *d*’ values observed for each material (used to calculate the *d*’% values presented in Figures 6, 7, 8) are shown in bold.

As can be seen in Table 4, the *d*’ values obtained for the five different rod materials and phantom sections range from values as high as 969 to values as low as 1.5, and therefore, it was very difficult to make comparisons among many different series using the original *d*’ values. To overcome this problem and be able to fit many series in the same graph and keep their variations visible to allow comparisons, the relative detectability index *d*’% values were used. These were derived by expressing the *d*’ values as percentages relative to the respective maximum *d*’ values obtained for each rod material. The maximum *d*’ values for air, water, bone, polystyrene, and iodine were 718, 94, 969, 45, and 358, respectively, and were all observed in the A‐4 image series, at the smallest diameter phantom section.

**FIGURE 6:**
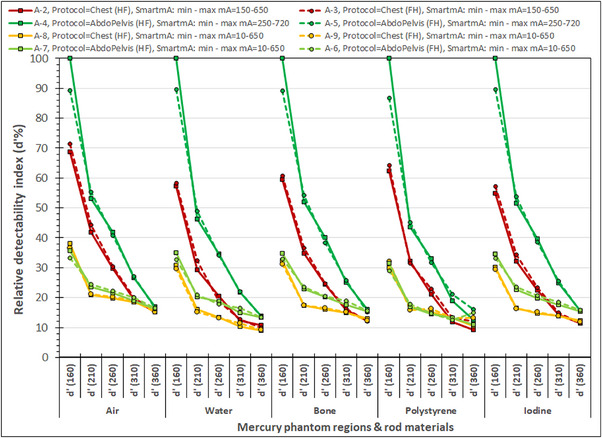
The relative detectability indices (*d’*%) for the different phantom sections and rod materials obtained using different acquisition protocols, scanning directions, and SmartmA minimum and maximum mA settings.

In Figure 6 are presented the relative detectability indices (*d*’%) for the different phantom sections and rod materials obtained using the Chest and AbdoPelvis acquisition protocols, both AP and FH scanning directions and both preset and modified SmartmA minimum and maximum mA settings. There are no considerable differences between the *d*’% values between the series performed with the same acquisition parameters and different scanning direction. For all series the *d*’ values decreased with increasing phantom section diameter. The AbdoPelvis protocol resulted always in bigger *d*’% values than the Chest protocol, when the preset SmartmA were used, but also when the modified SmartmA settings were used with only a few exceptions. It was striking, that though the DLP of the A‐7 and A‐8 series performed with the AbdoPelvis protocol and modified SmartmA settings, was almost half of that of the A‐2 and A‐3 series performed with the Chest protocol and preset SmartmA settings, they presented larger *d*’% values in all rod materials (except air) for the 310 and 360 mm diameter phantom cylinders.

**FIGURE 7:**
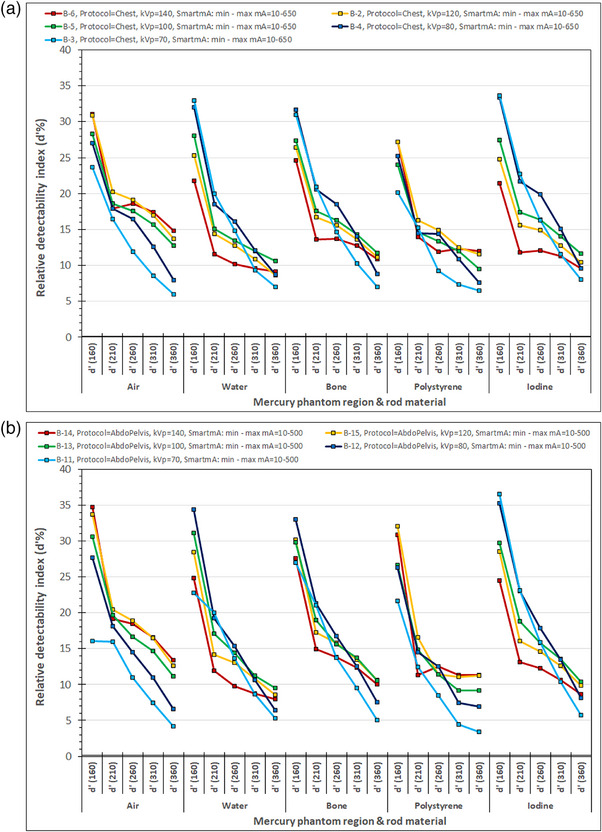
(a) The relative detectability indices (*d’*%) for the different phantom sections and rod materials obtained using the Chest acquisition protocol, and different manual kVp settings. (b) The relative detectability indices (*d’*%) for the different phantom sections and rod materials obtained using the AbdoPelvis acquisition protocol, and different manual kVp settings.

In Figure 7(a) where the *d*’% values obtained with the chest protocol and the modified SmartmA settings for various kVp values are shown, it can be seen that *d*’% values for 80 kV outscored the respective values of higher kVp selections in Water, Bone, and Iodine in all phantom sections but the 360 mm diameter. For these rod materials, the 70 kV selection produced similar or even larger *d*’% values than 80 kVp for the 160‐ and 210‐mm phantom sections, but the *d*’% values were reduced at the 260 mm sections, dramatically for the bone and iodine rods. In Figure 7(b) where the *d*’% values are shown for the AbdoPelvis protocol, similar observations were made with Figure 7(a), though *d*’% values for 100 kV produced similar or slightly larger *d*’% values than 80 kV for the 310 mm diameter section. In both Figure 7(a) and (b), it can be seen that for the air and polystyrene rods the *d*’% values for 120 kV in most cases and of 140 in the rest, outscored those that correspond to lower kVp selection but at a cost of a larger dose (about double or more) compared to 80 and 70 kVp. This is probably the reason that for both Chest and AbdoPelvis protocols with both the preset and modified SmartmA settings, the 80 kV was the automatically selected tube potential by the CT scanner.

**FIGURE 8:**
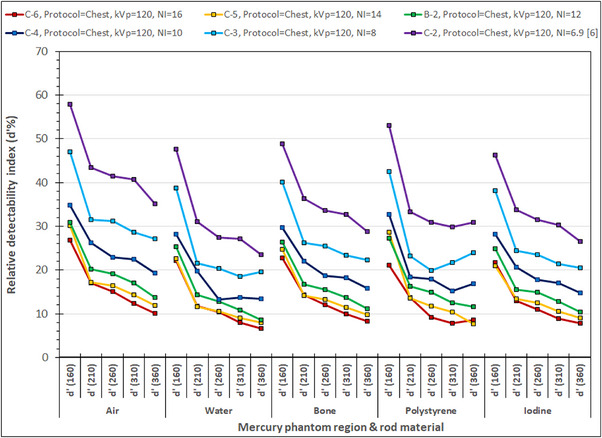
The relative detectability indices (*d’*%) for the different phantom sections and rod materials obtained using the Chest acquisition protocol and different manual NI settings.

In Figure 8 are shown the *d*’% values obtained with the Chest protocol, the modified SmartmA settings and different NI selections. It can be seen that with the exception of the curve for NI = 14, that overlaps in some cases with those for N‐12 and NI‐16, there is a distinct increase of *d*’% values with decreasing NI, for all phantom sections and all rod materials. It is thus clearly shown that *d*’ values increase with decreasing NI, when all other scanning parameters are kept constant, because of the dose increase.

**FIGURE 9:**
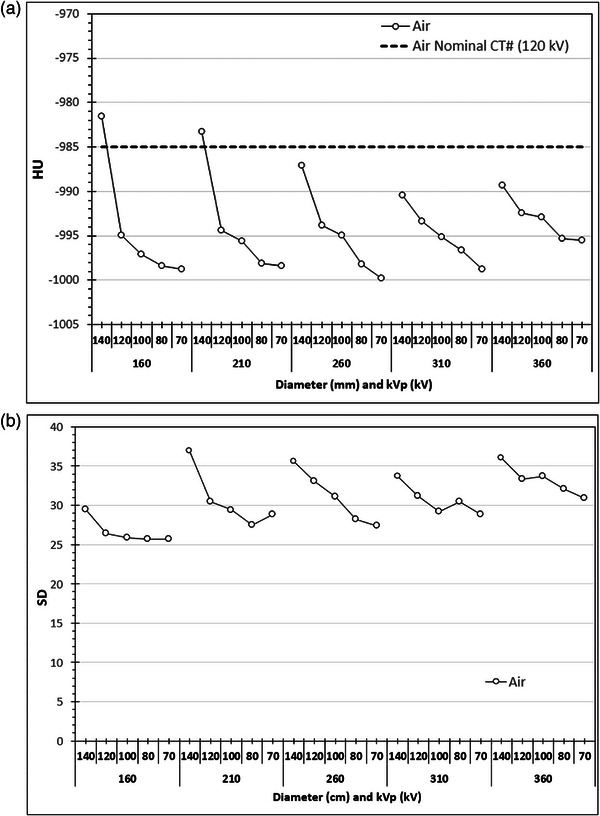
The variation of (a) CT# and (b) noise (SD), for the air rod at the different phantom sections obtained using the Chest acquisition protocol and different kVp settings (series B2‐B6: NI = 12).

**FIGURE 10:**
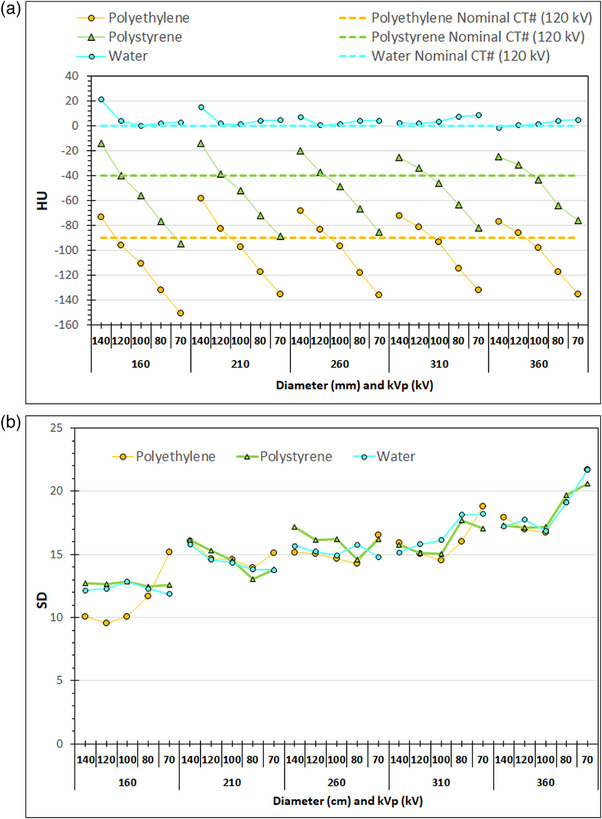
The variation of (a) CT# and (b) noise (SD), for the water and polystyrene rods at the different phantom sections obtained using the Chest acquisition protocol and different kVp settings (series B2–B6: NI = 12). For polyethylene the results are the average values from the 5 ROIs positioned within the rod materials but measured within the uniform sections of the phantom.

**FIGURE 11:**
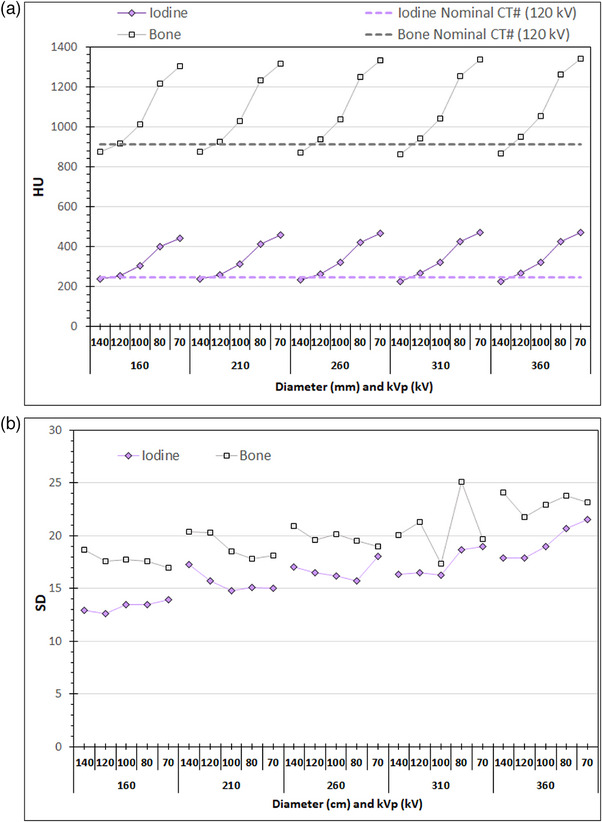
The variation of (a) CT# and (b) noise (SD), for the Iodine and Bone rods at the different phantom sections obtained using the Chest acquisition protocol and different kVp settings (series B2–B6: NI = 12).

Finally in Figures 9, 10, 11 are presented the average CT numbers (HU) and noise (SD) values derived with ImageJ at those CT slices that were used for the image quality evaluation with ImQuest, for the different rod materials and the different phantom sections, as a function kVp selection (Chest examination protocol, series B2–B6). As seen in Figure 9(a), the variation of HU values for the air rod is small but it follows a distinct pattern: it reduces with lower kVp, and also reduces with increasing phantom section diameter up to 310 mm. In Figure 9(b) the SD also reduces with reducing kVp selection, being smaller for the 160 mm diameter section and slightly increasing for all other phantom sections. In Figure 10(a), the variation of HU values for the water rod is generally small (slightly larger for 140 kV), but for the polystyrene rod and the polyethylene (phantom's main material) is large and follows a distinct pattern: for both materials HU is greatly reduced with lower kVp. In Figure 10(b) a gradual increase with phantom diameter is observed regarding SD for all materials. In Figure 11(a), the variation of HU values for the bone and iodine rods is large and follows a distinct pattern: for both materials HU is greatly increased with lower kVp, in contrast to what was observed for air, polyethylene, and polystyrene. In Figure 11(b) a gradual increase of SD with phantom diameter is observed for both iodine and bone.

## 4 DISCUSSION

Summarizing the main results presented in the previous section: the scanning direction did not have any effect on ATCM curves, but the scanning mode and beam width did both affect the ATCM curves. However, the SmartmA limit settings and the kVp selection had the greatest impact on image quality and dose. Indeed, it was seen that improper SmartmA limit settings can practically invalidate the ATCM operation. As can be seen in Table 3, with the preset SmartmA limit settings the DLP was 1.74 and 2.5 times the DLP values that resulted when using the modified SmartmA settings, respectively for the Chest and AbdoPelvis protocols. It must be noted that when selecting a minimum mA value for the SmartmA settings of an examination protocol, the rotation time used in this protocol must also be taken into account in order to set practically the desired minimum mAs limit (the same applies for the maximum mAs limit). For example, for the Chest protocol, where the minimum mA was 150 and the rotation time 0.6 s, the minimum mAs fore scan series A‐2 and A‐3 was 90, whereas for the AbdoPlevis protocol where the minimum mA was 250 and rotation time is 0.8 s, the minimum mAs for the scan series A‐8 and A‐7 was 200. When the modified SmartmA settings were used, the mAs values observed in the five increasing phantom diameters (at the rods’ positions) were respectively roughly equal to 16, 16, 28, 65, 124 (Chest series A‐8 and A‐9) and 12, 27, 43, 101, 191 (AbdoPelvis series A‐7 and A‐6). In the first set of mAs numbers only 124 is larger than 90, and this is why as can be seen in Figure 1, no modulation occurred till the second half of the 310 mm diameter phantom section for Chest series A‐2 and A‐3, whereas for AbdoPelvis series A‐4 and A‐5, no modulation at all was observed, since all the numbers of the second set are smaller than 200. If for both protocols a preset rotation time of 0.35 s were used, the respective minimum mAs values would be 52.5 for the Chest and 87.5 for the AbdoPelvis protocols, and then for both protocols modulation would occur in the phantom sections with diameters larger than 260 mm.

When the mA limitations were lifted using the modified SmartmA settings, it was seen that ATCM partially compensated for the steep decrease in image quality that occurs with increasing thicknesses when using practically constant mAs (see series A‐4 and A‐5 in Figures 1 and 6), as the *d*’% values slightly decrease with phantom thickness for all kVp settings. The gradual increase of noise with increasing phantom section diameter shown in Figures 9(b), 10(b), and 11(b), is practically in line with Figures 6, 7, 8, as they all suggest that image quality slightly reduces with increased phantom diameter. However, in Figures 6, 7, 8 the decrease seems to be more rapid, because apart from the noise increase with phantom diameter the losses in signal intensity within the five rod materials, should be also taken into account.^13^ This means that for this CT scanner the ATCM strategy is not to keep the image quality constant for all patient thicknesses at the expense of a large dose increase, but to balance the image quality and patient dose. Finally, an impressive dose reduction was observed with lower kVp selections, that for water, bone, and iodine materials was accompanied by improved *d*’% values in all but the largest sections of the phantom. Indeed, for the Chest and AbdoPelvis protocols the DLP reduces by about 20%, 46%, and 65% when using 100, 80, and 70 kV, respectively, and increases by about 12% when using 140 kVp.

Regarding the kVp selection, the results of this study are line with many studies with actual patients or phantoms that suggest the use of lower kVp (80–100) as a dose reduction strategy, that especially for examinations with iodine contrast media has proved not to compromise image quality.^13^, ^14^, ^15^, ^16^, ^17^, ^18^, ^19^, ^20^, ^21^, ^22^ For example, Stocker et al.^13^ who studied the impact of various dose reduction strategies in 3725 patients that underwent cardiac CT angiography for coronary artery evaluation concluded that the use of low potential (100 kV or less) is an efficient strategy to reduce radiation dose without compromising image quality. They also stated that low kVp techniques are underused in clinical practice. Kim et al.^19^ compared two groups of patients which underwent CT urography (CTU). In one group CTU examinations were performed using low kVp, low contrast concentration, and IR (LVLC‐CTU group: 80 kV, 240 mgI/mL and IR), while in the other were performed using high kVp, high contrast concentration, and no IR (conventional CTU group: 120 kV, 350 mgI/mL contrast, and filtered‐back projection). They concluded that the diagnostic acceptability and quantitative image quality of LVLC‐CTU with IR are not inferior to those of conventional CTU. Additionally, LVLC‐CTU with IR is beneficial because both radiation exposure and total iodine load are reduced. Regarding unenhanced studies, Choi et al.^20^ who studied 165 potential liver donors using unenhanced CT for non‐invasive diagnosis of hepatic steatosis, concluded that low‐kVp (100 kV) unenhanced CT is a robust technique with reduced radiation exposure for diagnosing moderate to severe hepatic steatosis. The non‐enhanced low‐kVp CT protocol showed similar to superior diagnostic performance to that of 120 kV CT. They also reported that reducing the tube potential from 120 to 100 kVp can reduce radiation exposure by about one third.

In our study, the activation of Auto kV mode reduced the kVp the chest protocol from 120 to 80 kV and for the AbdoPelvis protocol from 100 to 80 kV. This means that Auto kV selected a low kVp despite that the larger phantom diameter had a WED of 35 cm. Spearman et al.^21^ who studied the effect of ATVS on radiation dose at computed tomography (CT) worldwide encompassing all body regions and types of CT examinations, using data from 86 centers and 164 323 unique CT studies, concluded that ATVS (which usually selects a kVp value lower than 120 kV) significantly reduces radiation dose across most, but not all, body regions and types of CT examinations.

**FIGURE 12:**
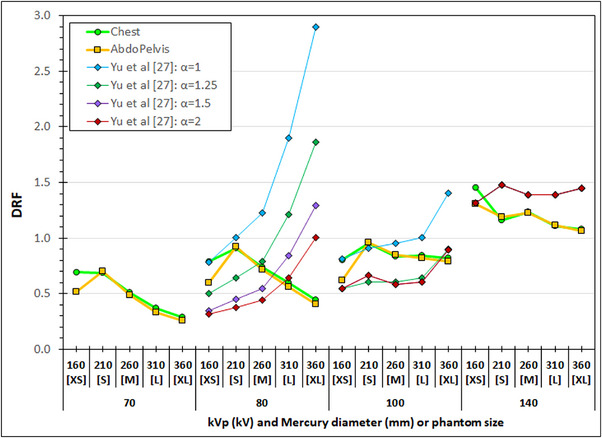
Comparison of the results of the current study (regarding the reduction of dose with decreasing kVp) with the results of Yu et al.^27^ The dose relative factor (DRF) is the ratio of the CTDI_vol_ value at the alternative kVp over the CTDI_vol_ value at the reference condition (120 kV). In the current study the CTDI_vol_ values were derived at the Z‐axis values of the five different cylindrical diameters of the Mercury phantom (at the middle of the rod sections), for the Chest (see Figure 3(a)) and AbdoPelvis (see Figure 3(b)) examination protocols. The DRF values from the Yu et al.^27^ were derived using five semi‐anthropomorphic thoracic phantoms with diameters 15, 30, 35, 40, and 48 cm, that were representing extra‐small (XS), small (S), medium (M), and extra‐large (XL) patients, using four different noise constraint (*α*) values. The DRF values for α = 1.5 and 2 in 100 kV, and all α's in 140 kV overlap.

The results of our study may seem to contradict the results of Yu et al.,^27^ who studied the automatic selection of tube potential as dose reduction a strategy in contrast‐enhanced CT examinations, using five semi‐anthropomorphic thoracic phantoms with diameters 15, 30, 35, 40, and 48 cm, that were representing extra‐small (XS), small (S), medium (M), and extra‐large (XL) patients, respectively. Considering 120 kV as the reference tube potential and dose, they determined what is the relative increase or decrease in dose when using 80, 100, and 140 kV, that provides the same or better contrast‐to‐noise ratio (CNR) for iodine as with the reference conditions, and the same or a little more noise. Therefore, CNR and noise were both used as surrogates and image quality, while CTDI_vol_ was used as surrogate of patient dose, and the relative dose factor (RDF) was defined as the ratio of CTDI_vol_ values at the alternative potentials and at 120 kV. Regarding the noise, the authors acknowledged that when the iodine CNR is the same or better, a relative increase in noise can be tolerated, depending on the diagnostic task, and they calculated different DRF values with noise constraints (*α*) of 1 (same noise), 1.25 (25% more noise), 1.5 (50% more noise), and 2 (100% more noise). The results of Yu et al.^27^ are presented in Figure 12 in comparison with the results of our study, using the underlying assumption that the five cylinders of the Mercury phantoms roughly correspond to the five phantom sizes used in the referenced study. It is obvious that there is a basic difference between the curves of Yu et al.^27^ and those derived with Mercury, which is more prominent at 80 kV. In Yu at al.^27^ the DRF increases with patient size for all diferent noise contraints, something that entails that the use of 80 kV presents large dose reductions primarily to thin and probably medium patients in cases where more noise can be tolerated. On the contrary, in our study it seems that the dose reductions obtained with lower kVp increase with patient size, since as seen the ATCM allows slightly more noise for the larger sections of the Mercury phantom. However, it must be emphasized that the study of Yu et al.^27^  was performed before the first iterative reconstruction (IR) algorithm for CT scanners was made commercially available, and therefore their results refer to the filtered backprojection reconstruction algorithm, in contrast to our results where IR (ASIR‐V) was used.

While the use of low kVp in CT exams is continuously gaining ground in clinical practice over 120 kV (or 140 kV), there are still many who insist on using 120 kV, probably because for so many years the use low kVp was not an option. It must always be remembered that it is the development of denoising algorithms such as those of IR, what have made it possible to perform CT at a low tube voltage setting without compromising diagnostic confidence, because they mitigate effects like photon starvation artifacts that occur at low kVp in larger patients or at low doses and reduce noise.^22^, ^23^, ^24^, ^25^, ^26^

However, a potential pitfall remains, and this is the change in CT number of all materials other than air and water (for which CT numbers are calibrated to remain roughly constant at all kVp selections). Though the change in CT numbers is the reason why the contrast of structures with both positive and negative CT numbers with respect to water increases, this change in CT numbers of tissues with or without contrast media may become important when a threshold or a range of CT numbers is used to discriminate normal from pathological tissues.^20^

## 5 CONCLUSION

While the use of the Mercury phantom was very helpful to understand many aspects of the ATCM operation, two are the most important findings of this study. First, failure to understand how the minimum and maximum mA selection work and what is their expected effect on clinical practice may practically incapacitate the ATCM system operation and greatly increase dose to slim patients. Second, lower kVp selections result in impressive dose reductions, which is combined in most cases with improvement (especially for iodine contrast enhanced studies) or in the worst case without considerable detriment of image quality, and the routine use of 120 kV should be probably reserved for obese patients only.

## AUTHOR CONTRIBUTIONS

All authors substantially contributed to the conception or design of the research and interpreted results. Data acquisition and initial analysis was made by Ioannis A. Tsalafoutas and Shady AlKhazzam. All authors contributed to drafting the manuscript or revising it critically for important intellectual content.

## CONFLICT OF INTEREST STATEMENT

The authors declare no conflicts of interest.
